# A Potential Link Between Oxidative Stress and Endothelial-to-Mesenchymal Transition in Systemic Sclerosis

**DOI:** 10.3389/fimmu.2018.01985

**Published:** 2018-09-19

**Authors:** Duong Thi Bich Thuan, Hatem Zayed, Ali H. Eid, Haissam Abou-Saleh, Gheyath K. Nasrallah, Arduino A. Mangoni, Gianfranco Pintus

**Affiliations:** ^1^Department of Biochemistry, Hue University of Medicine and Pharmacy, University of Hue, Hue, Vietnam; ^2^Department of Biomedical Sciences, College of Health Sciences, Qatar University, Doha, Qatar; ^3^Department of Pharmacology and Toxicology, Faculty of Medicine, American University of Beirut, Beirut, Lebanon; ^4^Department of Biological and Environmental Sciences, College of Arts and Sciences, Qatar University, Doha, Qatar; ^5^Biomedical Research Center, Qatar University, Doha, Qatar; ^6^Department of Clinical Pharmacology, College of Medicine and Public Health, Flinders Medical Centre, Flinders University, Adelaide, SA, Australia

**Keywords:** Endothelial-to-Mesenchymal Transition, oxidative stress, reactive oxygen species, scleroderma, systemic sclerosis

## Abstract

Systemic sclerosis (SSc), an autoimmune disease that is associated with a number of genetic and environmental risk factors, is characterized by progressive fibrosis and microvasculature damage in the skin, lungs, heart, digestive system, kidneys, muscles, joints, and nervous system. These abnormalities are associated with altered secretion of growth factor and profibrotic cytokines, such as transforming growth factor-beta (TGF-β), interleukin-4 (IL-4), platelet-derived growth factor (PDGF), and connective-tissue growth factor (CTGF). Among the cellular responses to this proinflammatory environment, the endothelial cells phenotypic conversion into activated myofibroblasts, a process known as endothelial to mesenchymal transition (EndMT), has been postulated. Reactive oxygen species (ROS) might play a key role in SSs-associated fibrosis and vascular damage by mediating and/or activating TGF-β-induced EndMT, a phenomenon that has been observed in other disease models. In this review, we identified and critically appraised published studies investigating associations ROS and EndMT and the presence of EndMT in SSc, highlighting a potential link between oxidative stress and EndMT in this condition.

## Introduction

Systemic sclerosis or scleroderma (SSc) is a complex multisystem autoimmune disease characterized by progressive fibrosis of the skin and visceral organs and significant vascular alterations (1). The pathogenesis of SSc remains unclear, particularly the mechanisms involved in the development of vascular lesions (2). Oxidative stress-mediated-vascular dysfunction and Endothelial-to-Mesenchymal Transition (EndMT) are likely to play a role in SSc-mediated vascular damage (3). Several studies have shown increased production of reactive oxygen species (ROS), altered redox state, and excessive extracellular matrix (ECM) deposition in organs and tissues of SSc patients (2). Although the primary cellular effector of diseases-associated fibrotic conditions remains to be identified (4) some potential sources have been proposed.

In addition to Epithelial-to-Mesenchymal Transition (EMT), which takes place *in vivo* in the lung (5–8), as well as in a variety of other fibrotic processes (9–13) including SSc (14, 15), the potential involvement of EndMT in SSc has also been suggested. EndMT accounts for the increased fibroproliferative vasculopathy and fibrosis in several diseases (16) and is considered a novel mechanism for the generation of activated myofibroblasts in SSc (17–20). On the other hand, increased ROS generation has been reported to mediate TGF-β-induced EndMT in several conditions including atherosclerosis, Fuchs endothelial corneal dystrophy, and diabetic nephropathy (21–23). TGF-β-mediated ROS generation also promotes cardiac fibroblast differentiation into myofibroblasts, which accounts for the increased production of ECM proteins such as type I and III collagen and the initiation of α-smooth muscle actin expression (α-SMA) during the EndMT process (24). Noteworthy, although not specifically in SSc, the regulation of TGF-β signaling by mitochondrial-derived ROS has also been reported in lung fibrosis (25, 26).

In this review, we summarize the most relevant research regarding the correlation between oxidative stress and EndMT, and their role in SSc-associated vascular damage and remodeling. Readers interested in a more comprehensive discussion concerning the mechanisms involved in the onset and progression of the fibrotic process can refer to other recent excellent reviews (27–30).

### Oxidative stress and SSc

The term ROS indicates oxygen-containing free radicals harboring one or more unpaired electrons in the atom or the outer molecular orbitals (31). Unpaired electrons make free radicals highly reactive. Among them, the superoxide radical (${\text{O}}_{2}^{\cdot -}$), hydrogen peroxide (H_2_O_2_), hydroxyl radical (^·^OH^−^), hypochlorous acid (HOCl) and peroxynitrite (ONOO^−^) are key oxidative molecules within the ROS family (32). In this regard, oxidative stress reflects an imbalance between the generation of ROS and the biological system's ability to counteract or detoxify their harmful effects. Therefore, when present in excessive concentrations, ROS cause oxidative stress and cellular damage, potentially leading to cell transformation and/or cell death (33).

ROS and oxidative stress are considered to play a key role in the onset and progression of SSc through several processes, such as ischemia-reperfusion injury (34–36). In addition, ligand-mediated receptor activation by cytokines and growth factors can also increase ROS generation (2, 35, 37). For example, TGF-β is a profibrotic cytokine that plays a key role in the ligand-mediated receptor process that triggers the onset and progression of SSc (2, 37–41). Other putative factors involved in the pathogenesis of SSc include the platelet-derived growth factors (PDGF), vascular endothelial growth factor (VEGF), connective tissue growth factor (CTGF), angiotensin II, interleukin 3, interleukin 6, tumor necrosis factor-alpha (TNF-α), nerve growth factor, and fibroblast growth factor (FGF). These factors can generate ROS in vascular smooth muscle cells, cardiac, lung and skin fibroblasts by activating signaling pathways coupled to nicotinamide adenine dinucleotide phosphate oxidase (NOX) family members (42). Cytokines can also modulate ROS generation by influencing the cellular concentrations of NOX at both RNA and protein levels, as well as by improving both stability and NOX translocation to the cell membrane by stimulating the phosphorylation of NOX complex's components (42). For instance, in rat aortic smooth muscle cells, production of ROS by IL-1β is mediated by NOX4 (43). Although IL1-β induces its expression (44), NOX1 does not appear to be involved in IL1-β-induced ROS production (43). In addition, TGF-β has been shown to induce NOX4 expression as well as ROS production in human arterial smooth muscle cells (45). IFN-γ, via the JAK/STAT pathway (46), and TNF-α, likely via the NFκB pathway (47), also induce expression of NOX1 and NOX4 in human aortic smooth muscle cells (48). Indeed, NOX inhibitors appear to attenuate the effect of TNF-α in vascular smooth muscle cells, supporting the hypothesis that this cytokine elicits its effects via NOX activity (49). The signaling pathways involved in cytokines-induced ROS generation in fibrosis and SSc are quite complex and depend on specific cytokines, NOX isoforms and target cells. For a more detailed information regarding this aspect we refer the readers to this excellent review (42) Furthermore, besides cytokines-induced ROS generation, the increased concentrations of superoxide from different cells, including fibroblasts and monocytes, can contribute *per se* to ROS elevation in SSc (2, 50–54).

About 90% of patients with SSc suffer from Raynaud's phenomenon, a condition where the cold-induced constriction of dermal arterioles is excessively augmented and results in vasospasm and skin color change. Patients with Raynaud's phenomenon secondary to underlying diseases typically present with more severe manifestations such as ulcer, scar, or gangrene (55, 56). Although the detailed molecular pathology of the Raynaud's phenomenon, and its association with SSc, is not clearly understood, both oxidative and non-oxidative pathways appear to be involved (35, 56–58). The systemic increase of ROS concentrations in SSc is likely to be an important factor for the worsening of the Raynaud's phenomenon. In this context, the concentrations of 8-isoprostane, a biomarker of oxidative stress, antioxidant deficiency and lipid peroxidation, have been shown to correlate with the extent of vascular lesions in Raynaud's phenomenon and the severity of fibrosis in patients with SSc (59–61). The free radical nitric oxide (NO), released by the endothelial cells, plays an essential role in the homeostatic control of vascular tone and blood pressure as well as in preventing thrombosis and cell damage. However, during the reperfusion phase in the Raynaud's phenomenon, free radicals and NO lead to peroxynitrite formation, which precedes oxidative vascular damage and endothelial apoptosis. Therefore, in this specific situation, NO further aggravates vascular damage (35, 62).

A growing number of *in vitro* and *in vivo* studies have demonstrated the direct role of ROS in the pathogenesis of SSc (61, 63, 64). Grygiel-Gorniak and Puszczewicz et al. skin and visceral fibroblasts from SSc patients spontaneously produce large amounts of ROS that initiate collagen synthesis (35). Indeed, fibroblasts from SSc patients have higher baseline NOX-inhibitable intracellular ROS concentrations (65) when compared to fibroblasts from healthy donors (65). This phenomenon appears to be triggered by the stimulation of the PDGF receptor and further maintained through ROS-ERK1/2 signals mediated by Ha-Ras (66). It is important to emphasize, however, that normal fibroblasts can also respond to stimulation by different cytokines with a NOX-dependent increase in intracellular ROS concentrations (65).

A preliminary study by Boin et al. (67) showed a significant increase in intracellular ROS concentrations in human pulmonary artery smooth muscle cells (HPASMCs) after treatment with sera from patients with SSc and pulmonary artery hypertension (PAH). NOX2ds-tat (gp91ds-tat), a specific inhibitor of NOX2, prevented the PAH-SSc sera -induced ROS generation, suggesting the mechanistic involvement of NOX2 in this phenomenon (67). Exposure of HPASMCs to SSc-PAH sera also resulted in a progressive increase of the Collagen promoter activity. Similarly, this effect was prevented by NOX2ds-tat treatment, suggesting that the collagen synthesis activation in HPAMSCs is driven by SSc-related PAH sera through NADPH oxidase-dependent ROS generation (67). Moreover, in human dermal fibroblasts, NOX inhibition caused a significant reduction in the expression of fibronectin, collagen type I and alpha-smooth muscle actin (68). Similarly, the selective NOX1/NOX4 inhibitor, GKT-137831, abolished TGF-β-induced expression of the profibrotic genes CCN2 and alpha-SMA (41). Importantly, GKT-137831 was also able to reduce collagen gel contraction as well as expression of alpha-SMA and CCN2 protein overexpression in fibroblasts isolated from dermal lesions in SSc patients (41). A NOX4-derived increase of ROS has also been reported to be involved in the vascular smooth muscle cells contractile to synthetic phenotype switch elicited by agonistic anti-PDGF receptor autoantibodies from SSc patients (69) Taken together, these findings indicate that the NOX system, in addition to ROS production, mediates the activation of collagen synthesis and profibrotic genes.

*In vivo* studies in BALB/c mice injected with HOCl daily for 6 weeks (a murine model of SSc) showed the induction of chronic oxidative stress and the concomitant development of cutaneous and lung fibrosis. Furthermore, HOCl-treated mice overexpressed α smooth muscle actin (α-SMA), a marker of myofibroblast activation. These processes were mediated by the ROS-activated intracellular signaling pathways ADAM17/Notch and Ras-ERK (70–72). Blockade of the Ras-ERK pathway by propylthiouracil (PTU) or simvastatin prevented both cutaneous and lung fibrosis (71, 72).

**Figure 1:**
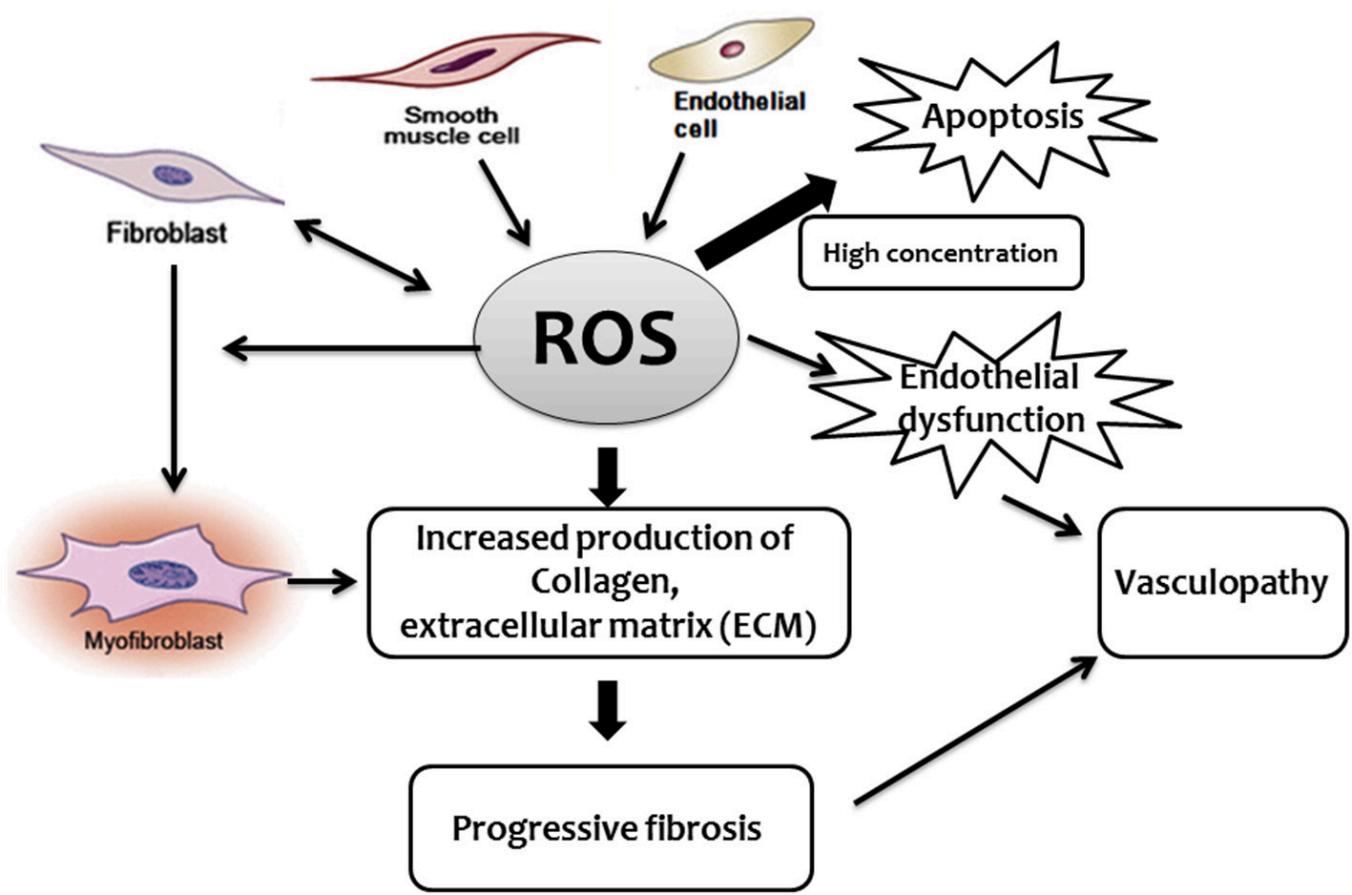
Schematic diagram describing the central role played by ROS in the development of fibroproliferative vasculopathy and fibrosis in SSc.

Some studies reported that, in SSc, the excessive production of ROS activates fibroblasts through the binding of stimulatory serum autoantibodies to the PDGFR (73). In this regard, it has been reported that sera from mice and patients with SSc induce fibroblast proliferation and H_2_O_2_ production by endothelial cells, phenomena that appear to be mediated by oxidized auto-antigens such as the oxidized DNA topoisomerase (74). Noteworthy, the same paper indicated that the nature of ROS, as well as the induction of different antibodies, appear to dictate the form of SSc in both mice and humans. BALB/c SCID mice treated with peroxynitrites developed skin fibrosis and serum anti centromere protein autoantibodies (anti-CENP-B), as reported in patients with limited cutaneous scleroderma, while mice treated with hypochlorite or hydroxyl radicals developed skin and lung fibrosis and DNA topoisomerase I autoantibodies, as reported in patients with diffuse cutaneous scleroderma (74). While pro-oxidants may cause an increase in autoantibodies, other studies failed to demonstrate a significant association between autoantibodies in endothelial cells and fibroblasts and serum-induced ROS or cell proliferation. Despite this uncertainty, there is good evidence supporting the role played by autoantibodies in SSc, particularly anti-endothelial cell antibodies (AECA), in the development of pulmonary fibrosis (35, 75). Oxidative stress may either directly activate ROS-induced differentiation of fibroblasts into myofibroblasts (70), disrupt the balance between protease and protease inhibitors, or both. TGF-β upregulates the expression of extracellular matrix proteins including collagens, but also suppresses protein degradation through enhancing the activities of protease inhibitors such as plasmin activator inhibitor 1 (PAI-1) and tissue inhibitors of metalloproteinases (TIMPs) (38). This protease-antiprotease imbalance is likely to represent a critical factor in the development of SSc lung fibrosis (76). Furthermore, although not specifically demonstrated in the SSc-associated fibrotic process, a mitochondrial-generated increase of ROS has been found to induce lung fibrosis (25–27). Effect of ROS in the development of fibroproliferative vasculopathy and fibrosis in SSc (Figure 1).

### Endothelial-to-mesenchymal transition and SSc

The pathogenesis of SSc involves several complex mechanisms associated with (i) microvascular fibroproliferative lesions, (ii) innate and adaptive immune system abnormalities and uncontrolled accumulation of collagen, as well as (iii) other extracellular matrix compartments produced by fibroblasts and activated myofibroblasts in the skin and other organs (17, 77). Physiologically, myofibroblasts die through apoptosis and/or the transition to a quiescent/senescent state in the late stages of wound healing. However, the persistence of activated myofibroblasts contributes to progressive fibrogenesis (78, 79) and favors the onset and progression of interstitial and perivascular fibrosis in the lungs, heart, kidneys and other organs, which accounts for the high mortality of SSc patients (80).

Myofibroblast activation in SSc has been demonstrated in pericytes and smooth muscle cells (SMCs) from vessel walls, resident fibroblasts, and bone marrow-derived fibroblasts (17, 81, 82). Although the ontogenesis of myofibroblasts in fibrotic conditions remains an area of active research (4, 83–86), an increasing number of studies indicated EMT as a potential source of activated fibroblasts by which epithelial cells transform into myofibroblasts (5–8, 14, 15). In this regard, some authors suggest that diseases-associated fibrotic processes may be the result of injury-elicited cellular stress responses such as senescence or apoptosis (28, 87–91), These processes, under yet unknown conditions, could also promote tissue repair by activating and/or recruiting resident progenitor cells (92) especially in the lungs (93–95). However, this hypothesis does not exclude the coexistence of other injury-activated processes such as the cellular transdifferentiation of endothelial cells to profibrotic activated myofibroblasts during EndMT, a phenomenon that has been reported also *in vivo* (19, 96–102). The latter might provide a plausible explanation for the excessive secretion of extracellular matrix proteins that takes place in this pathological condition (103). EndMT is considered a distinct form of EMT since vascular endothelial cells share several similar characteristics and molecular mechanisms with epithelial cells in generating fibroblasts and myofibroblasts (104, 105). EndMT contributes to the development of cardiac, pulmonary, renal, liver, and intestinal fibrosis, and idiopathic portal hypertension in SSc (7, 12, 13, 17, 18, 97, 98, 106–109). EndMT has also been reported *in vivo* in pulmonary hypertension, a process that is closely associated to SSc (101, 102, 110). It is also important to mention that, despite its role in many pathologies, EndMT may be beneficial during angiogenic sprouting, as it allows cells to lose intercellular junctions and delaminate from the parent vessel (111).

During the conversion of endothelium into mesenchyme elicited by TGF-β or Notch ligands, endothelial cells undergo morphological alterations and loss of characteristic cell-surface markers, acquiring mesenchymal, fibroblast-like properties such as spindle-shaped morphology, migratory capacity, invasiveness, and enhanced resistance to apoptosis (82, 112). During EndMT, the structure of vessel-lining is disrupted due to resident endothelial cells disaggregating from the organized cells layer in the vessel walls and invading the surrounding tissue (112–114). Cell-surface markers such as vascular endothelial cadherin (VE-Cadherin), CD31 (platelet endothelial cell adhesion molecule-1, PECAM 1), and von Willebrand factor (vWF) are gradually replaced by markers such as fibroblast-specific protein-1 (FSP-1), α-SMA, vimentin and type I and type III collagen (12, 18, 114).

Several studies have demonstrated the presence of transitional EndMT cells in the pulmonary vasculature of patients with SSc-PAH, indicating their possible contribution to vascular remodeling and fibrosis (18). For instance, the coexpression of cell surface markers specific for both endothelial and mesenchymal/fibroblastic cells, such as Willebrand factor (vWF) and α-SMA, has been reported in an experimental murine model of PAH and in pulmonary endothelium samples of SSc-PAH patients (110). Similar findings have been reported in lung tissue from patients suffering from SSc-associated interstitial lung disease (19). Cells in the intermediate stages of EndoMT have also been found in dermal vessels from patients with SSc and in two types of SSc animal models, bleomycin-induced SSc and urokinase-type plasminogen activator receptor (uPAR)-deficient mouse model (115). When compared to normal skin microvascular endothelial cells from healthy donors, cells from SSc patients displayed a spindle-shaped figure along with co-expression of both endothelial (CD31 and VE-cadherin) and myofibroblast markers (α-SMA, S100A4, type I collagen). Moreover, exposure of healthy donor-derived microvascular endothelial cells to either SSc sera or TGFβ1 triggered the transition to a myofibroblast-like morphology, contractile phenotype, downregulation of endothelial markers and induction of mesenchymal markers (115). Similarly, exposure of pulmonary artery endothelial cells to a mixture of proinflammatory cytokines such as IL-1β, TNF-α, and TGF-β abolished their “cobblestone” structure and prompted a spindle-like appearance along with induction of mesenchymal markers (110). Induced endothelial-mesenchymal transition (I-EndMT) cells exhibit an increased secretion of proinflammatory proteins and collagen type I. In addition, the presence of I-EndMT cells in the cellular barriers leads to a significant increase in paracellular and transcellular permeability, an early sign of vascular dysfunction in SSc (76, 110).

Vascular abnormalities in SSc patients have been reported to affect the structure and function of several organs and systems, such as the kidney, lung, skin, heart, gut, penis and large vessels (17). Therefore, the mechanistic contribution of EndMT to the pathogenesis of SSc vasculopathy is postulated to involve a synergistic and complex activation of a large variety of endothelial cell types from different body districts exposed to multiple local biological mediators, particularly TGF-β, but also IL-1β, TNF-α, PDGF, VEGF, and endothelin-1 (ET-1) (17, 18, 77, 78, 112, 114, 116–119). Indeed, cytokines-mediated receptor activation induces the expression of endothelial cell adhesion molecules such as ICAM, VCAM-1, and E-selectin (120, 121), which promote both recruitment and activation of chronic inflammatory cells, such as T- and B-lymphocytes and profibrotic macrophages, in the perivascular tissue and in the parenchymal organs interstitium. Recruited chronic inflammatory cells secrete transforming TGF-β, CTGF, and other profibrotic growth factors which along with endothelial cell released mediators, such as endothelin-1, enhance the fibroproliferative vasculopathy characteristic of the disease and potentially activate EndMT (17, 122, 123). In this regard, the interaction between proinflammatory stimuli and endothelial cells has been demonstrated by a recent paper reporting the upregulation of ET-1 and TGF-β in human microvascular endothelial cells induced by IF-γ, (118). Nevertheless, the precise molecular mechanisms by which inflammatory cytokines, growth factors, and signaling pathways mediate EndMT are not completely elucidated and necessitate further investigation.

Using a transgenic mice model, a recent study demonstrated that the endothelial cell-specific activation of TGF-β1 downstream signaling pathway induces EndMT in lung vessels (20). An elegant study using co-culture of microvascular endothelial cells and fibroblasts isolated from the skin of SSc patients demonstrated the ability of SSc-fibroblasts to induce endothelial cells EndMT transition. Consistent with this finding, normal fibroblasts treated with TGF-β and ET-1 were able to promote the same effect when co-cultured with microvascular endothelial cells (124). TGF-β2 has also been reported to mediate Interferon-γ-induced EndMT in human dermal microvascular endothelial cells (118). Protein kinase C has also been reported to be involved in TG-induced EndMT of mouse pulmonary endothelial cells *in vitro* (125).

All TGF-β isoforms have been reported able to elicit EndMT although the precise role of each isoform appears to differ between species and requires further studies to be clearly elucidated. While TGF-β 1 appears to be the main isoform involved in fibrosis-associated EndMT processes, TGF-β 2 seems to be primarily involved in EndMT associated to embryonic heart development (126–128). Nevertheless, also TGF-β 2 has been recently reported to be involved in EndMT associated with some pathological process such as cardiac hypertrophy and renal fibrosis (129, 130). In this regard, Maleszewska et al. (131) showed that TGF-β can either promote EndMT *per se* or synergize with TNF-α and IL-1β to induce the transdifferentiation of endothelial cells toward the profibrotic activated myofibroblasts phenotype. The authors showed that, in the framework of an inflammatory co-stimulation, TGF-β 2 is more potent that TGF-β1 in inducing EndMT, suggesting that TGF-β 2 may be the primary EndMT trigger, while IL-1 is necessary for the efficient induction of EndMT, but is not essential for its maintenance (131). The finding that endothelial cells in proinflammatory environment respond differently to TGF1 and TGF2 suggests that, similar to what reported in the embryonic development, TGF2 might play a major role in EndMT associated to pathological inflammation. In this regard, Good et al. (110) showed that a combination of TGF-β with IL-1β and TNF-α induced EndMT in pulmonary artery endothelial cells. Notably, the withdrawal of IL-1β, TNF-α, and TGF-β after 6 days failed to revert this process, suggesting that the phenotypic change might be permanent (104, 110). This observation was in agreement with previous studies in cells exposed to activated Ras and TGF-β treatment (107). Recently, Cipriani et al. (119) reported that Macitentan, an Endothelin-1 Receptor Antagonist, blocks both Endothelin-1- and TGF-β-induced EndMT in microvascular endothelial cells isolated from healthy donors and SSc patients (119). Similar results, using both Bosentan and Macitentan, were also obtained by Corallo et al. (124) in the fibroblast and microvascular endothelial cells coculture model previously discussed (124). Other studies reported that some anti-fibrotic mediators could dedifferentiate established myofibroblasts. These findings suggest the possibility that EndMT is a reversible process, providing a new intriguing therapeutic target for fibrotic diseases (81, 132).

**Figure 2:**
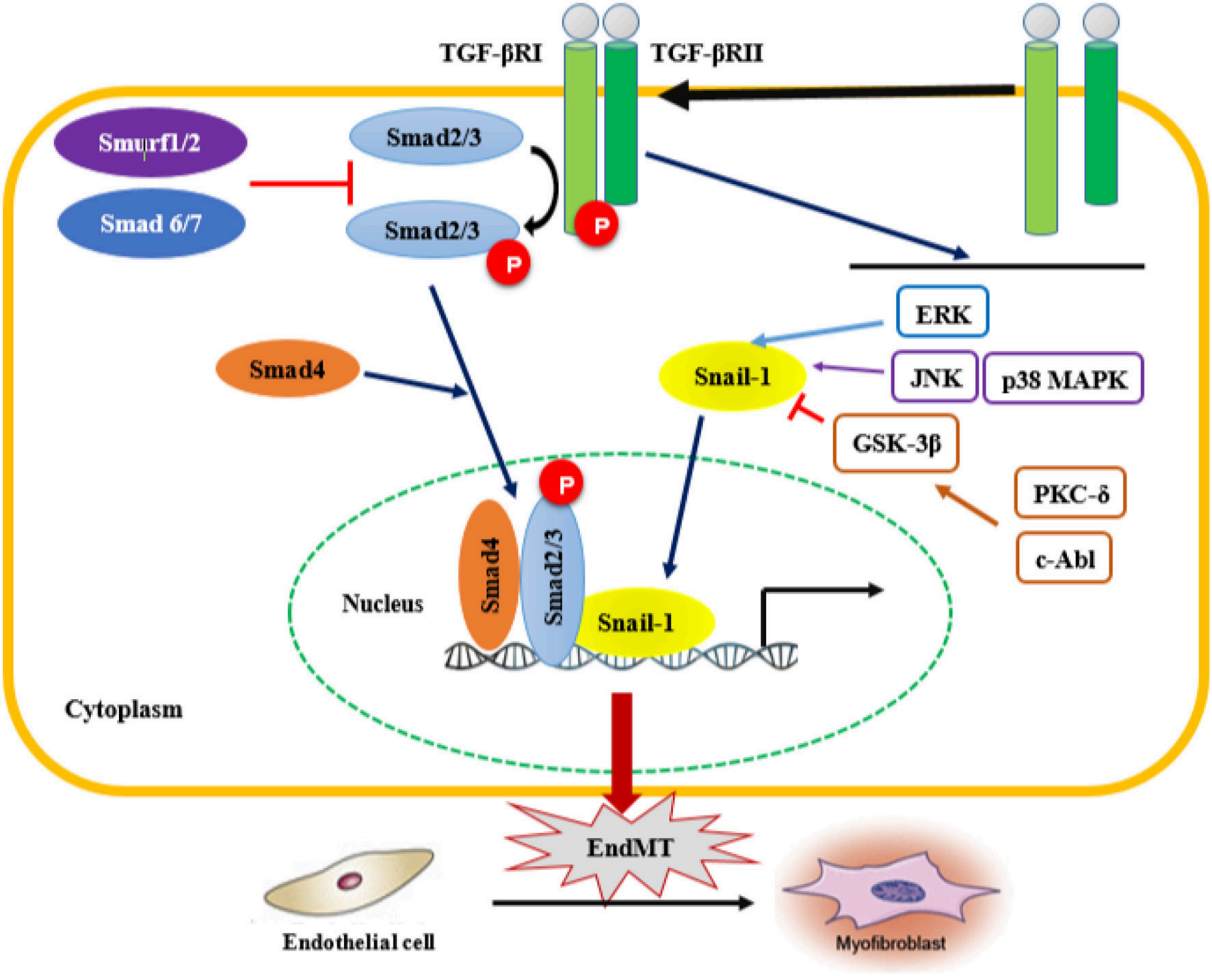
Smad-dependent and Smad-independent pathways of TGF-β signaling associated with EndMT. Transforming growth fator-β (TGF-β) signaling activates the downstream signal transduction cascades, Smad and non-Smad pathways. TGF-β binds the TGF-β type II receptor (TGF-βRII), which recruits and activates the type I TGF-β receptor. TGF-βRI in turn phosphorylates Smad2/3, which forms a complex with Smad 4. In addition, TGF-β activates Smad-independent pathways. Activation of Smad-independent TGF-β pathway causes phosphorylation of GSK-3β mediated by PKC-δ and c-Abl. Phosphorylation of GSK-3β causes its own inhibition which then allows Snail-1 to enter the nucleus. TGF-β/Smad-dependent and Smad-independent pathways upregulate the transcription of TGF-β target genes such as α-smooth muscle actin (α-SMA), fibronectin and type I collagen, as well as the transcription factor Snail-1 involved in EndMT. EndMT leads to the transdifferentation of ECs into mesenchymal cells, which subsequently transform into myofibroblasts, therefore contributing to the progression of fibrotic diseases. ERK, extracellular signal-regulated kinase; JNK, jun N-terminal kinase; p38 MAPK, p38 mitogen-activated protein kinases; PKC-δ, protein kinase C δ; c-Abl, c-Abl protein kinase; GSK-3β, Glycogen synthase kinase 3β.

TGF-β plays a critical role in other signaling pathways involved in EndMT, such as the Smad-dependent and Smad-independent pathways (Figure 2). Smad proteins have been shown to bind directly to the Snail gene promoter and regulate its transcription (117). Snail-1 is a zinc-finger transcription factor that forms a complex with Smad3/Smad4, and by acting as transcriptional repressor, plays a crucial role in the TGF-β-induced mesenchymal transdifferentiation of embryonic stem cell-derived ECs. The active Smad3/Smad4/Snail-1 complex is a potent inhibitor of E-cadherin expression by directly integrating into specific sequences within the gene promoter and blocking its transcription. In addition to E-cadherin inhibition, Snail-1 precedes transcriptional events that lead to the expression of a mesenchymal-cell-specific phenotype (17, 18).

In the Smad-independent pathway of TGF-β signaling, there is an involvement of important kinases such as c-Abl protein kinase (c-Abl), protein kinase Cδ (PKC-δ), RhoA and glycogen synthase kinase 3β (GSK-3β). The function of RhoA in actin and microtubule cytoskeleton organization is well-established, and as such contributes to the structural/phenotypic changes observed during EndMT (133). On the other hand, activation the of Smad-independent TGF-β pathway causes GSK-3β inhibition allowing Snail-1 to enter the nucleus. Indeed, phosphorylation of GSK-3β at the specific Ser9 residue causes its own inhibition, which in turn induces Snail-1 up-regulation and promotes its subsequent translocation into the nucleus. This process increases the nuclear accumulation of Snail-1, which consequently drives the expression of mesenchymal cell-specific markers such as α-SMA and type I collagen, while reducing the expression of VE-cadherin harbored in endothelial cells. Inhibitors of c-Abl and PKC-δ, such as imatinib and rottlerin, counteract the phosphorylation of GSK-3β, which allows GSK-3β to phosphorylate Snail-1, targeting it for proteasomal degradation and ultimately abolishing the transdifferentiation of ECs into myofibroblasts. This intervention could be a therapeutic strategy to counteract the acquisition of the myofibroblastic phenotype during EndMT (125). TGF-β2-downstream signals mediated by MEK, PI3K, and p38MAPK pathways are also essential for ECs undergoing EndMT transition (117). In addition, regulation of EndMT by Wnt (134), NOTCH (135), and Caveolin-1 signaling (136, 137) has been observed prior to the TGF-β-induced endothelial-mesenchymal transition. ET-1-mediated TGF-β1-induced EndMT has been also confirmed in skin and lungs *in vivo* in an animal model of TGF-β1-induced tissue fibrosis (138). Finally, other cytokines and growth factors such as PDGF, VEGF, ET-1, CTGF, and some MicroRNAs (miRNAs) might also be involved in the endothelial-mesenchymal transition (17, 18).

miRNAs are small non-coding RNAs containing about 22 nucleotides, which are post-transcriptional repressors of gene function (139). miRNAs have been recently reported to play important roles in SSc pathogenesis as well as in the EndMT process (140, 141). However, further studies are required to determine whether the SSc-associated profibrotic or antifibrotic effects of specific miRNAs are mediated by EndoMT. Recent studies reported the involvement of miRNAs 125b and 126 in the development of EndoMT (142–144) and the interaction between TFG β and several miRNAs in modulating EndMT. For instance, miRNA21 partially mediated TGF-β-induced EndoMT (145, 146). miR-155 is higly overexpressed during the EndoMT process and potently inhibits TGF-β induced EndoMT through a mechanism involving RhoA signaling (147). The overexpression of miR-148b increased EC migration, proliferation, and angiogenesis, whereas its inhibition promoted TGF-β-induced EndoMT (148). On the other hand, other studies indicated some miRNAs as positive modulators of TGF-β-induced EndoMT. For instance, the constitutively expression of miR-31 positively regulates TGF-β-induced EndMT in cultured endothelial cells (149). Interestingly, the overexpression of miR-130a, which is upregulated in monocrotaline-induced the Pulmonary arterial hypertension (PAH) mouse model, can elicit the expression of α-smooth muscle actin, a critical component in EndMT transition and fibrogenesis (150). Since PAH is a widely SSc-associated condition, this paper my pave the way for further experimentations in this direction. Many other miRNAs have also been reported to be involved in EndoMT modulation including let-7 (151, 152) and miRNA 29s (153). Given the pivotal regulatory effects exerted by miRNAs on the multitude of signaling pathways involved in the pathophysiology of multiple diseases, we expect that the knowledge regarding the putative contribution of miRNA to the EndMT process in conditions such as SSc will rapidly expand providing valuable information both to unravel the EndoMT mechanistic processes and to identify potential therapeutic targets for fibrotic disorders in general.

### Reactive oxygen species and endothelial-to-mesenchymal transition

ROS have been proposed as key mediators of EMT in renal tubular epithelial cells, human epithelial keratinocytes, and lung epithelial cells (32, 105, 154–156). An emerging issue is whether there is a similar tendency of endothelial cells exposed to oxidative stress to form transitional EndMT cells. Although EMT and EndMT share several similarities, in terms of signaling pathways and outcome, the two processes need to be differentiated due to the various origin, functions, and microenvironment of epithelial and endothelial cells (157, 158). Whether EndMT is a reversible biological process, similar to EMT (157), is an intriguing question that deserves further investigations.

### ROS activate/mediate TGF-β-dependent signaling pathways in EndMT

TGF-β is a multifunctional protein, including three isoforms (TGF-β1, TGF-β2, and TGF-β3), which regulates several important physiological processes such as cell proliferation, differentiation, apoptosis, adhesion, and migration (38, 159). However, a critical, potentially vicious, cycle of TGF-beta, and ROS interaction exists. For instance, TGF-β stimulates the generation of ROS in various cells while ROS can activate TGF-β and mediate its effects. Moreover, TGF-β elevates ROS production via NOX4, mitochondria, or microsomes, and ROS, in turn, can induce TGF-β gene expression and activate its signaling through oxidizing latency association protein (LAP) or activating MMPs which promotes LAP release (38). It is well known that TGF-β is synthesized as a non-active pro-form combining with LAP to create a latent complex. The ROS-oxidable redox center present in LAP can trigger a conformational change resulting in the release of TGF-β1 (160). Hence, under the stimulation of ROS, TGF-β is activated and increasingly expressed.

It is well documented that TGF-β induces EndMT in cardiac, pulmonary, renal, intestinal, and skin tissues (12, 106, 107, 161, 162). In this context, NADPH oxidases and ROS play a pivotal role in mediating TGF-β induced fibrotic responses via Smad2/Smad 3 activation (159). Of note, NOX4-dependent generation of H_2_O_2_ is required for TGF-β1-induced myofibroblast differentiation and ECM production (163, 164). A recent study by Montorfano et al. (32) used human umbilical vein endothelial cells (HUVECs) to investigate the role of ROS as well as the underlying mechanism in the conversion of ECs into myofibroblasts (32). The authors demonstrated that oxidative stress is a crucial factor in generating the EndMT phenotypic conversion of ECs via TGF-β1 and TGF-β2-dependent pathway and that the ALK5/Smad3/NF-κB intracellular pathway mediated the observed phenomenon. This study supports the hypothesis of an interaction between ROS, TGF-β, and EndMT. Indeed, increased ROS prompted the expression and secretion of TGF-β1/2, with consequent activation of its downstream signals, while silencing of TGF-β1/2 abolished the oxidative stress-induced conversion (32).

Another recent study by Echeverría et al. (165) provided evidence that lipopolysaccharide-induced ROS could lead to an EndMT-like process through an ALK5 activity-dependent mechanism (165). In line with this observation, Toshio et al. (109) showed that endotoxemia-derived oxidative stress promotes TGF-β-mediated EndMT in pulmonary vascular endothelial cells (109). This evidence was strongly supported by another study showing that the expression of TGF-β1 and TGF-β2 is crucial for the development of endotoxin-induced endothelial fibrosis (166). The increase in endotoxin-induced TGF-β1 and TGF-β2 expression required the activation of NOX and the subsequent generation of ROS (166). Collectively, these data suggest that oxidative stress mediates the EndMT process induced by TGF-β.

### Oxidative stress and EndMT in SSc: is there a link?

Despite the fact that the relationships between ROS and SSc, and between EndMT and SSc, have been extensively investigated, relatively few studies have investigated the effects of oxidative stress on EndMT in SSc. Xu et al. (167) showed that chronic oxidative stress mediates EndMT in a murine model of SSc (167). The authors isolated microfibrils from the skin of tight skin (Tsk^+/−^) mice, which showed abnormal big fibrillin-1. Culturing ECs with this abnormal extracellular matrix led to morphological and functional cellular changes, and increased the concentrations of 4-HNE, a well-known fission product of polyunsaturated fatty acid oxidation. Transdifferentiation from ECs to mesenchymal cells with the increased presence of FSP-1 and Twist (a transcription factor implicated in the endothelial cell to fibroblast transition), along with the decreased expression of VE-cadherin, were also noted (167). Furthermore, the abnormal big fibrillin expression was associated with oxidative stress (reduced nitric-oxide-to-superoxide anion ratio) suggesting changes in the intracellular redox state involved in the observed transition. Interestingly, chronic mice pretreatment with D-4F, a peptide binding with high affinity to oxidized lipids, attenuated or abolished EndMT, indicating that oxidized lipids might play a central role in other chronic conditions where oxidative stress promotes endothelial-mesenchymal transition (167). However, the authors did not dissect the role of TGF-β although, as mentioned above, TGF-β is known to be a crucial player in the initiation of EndMT in various diseases (17, 18). Of note, ECM including fibrillin functions as a reservoir for TGF-β and other growth factors to control mesenchymal differentiation (168). The possibility of an interaction between TGF-β and oxidative stress prior to EndMT is intriguing and requires further research.

It is speculated that the local availability of tetrahydrobiopterin (BH_4_) contributes to endothelial physiology (169), and that its insufficiency might be involved in the endothelial dysfunction observed in SSc (170). Decreased concentration of BH_4_ induces eNOS uncoupling, leading to superoxide, rather than NO, production, which in turn induces a state of oxidative stress (171). Although not able to reverse blood oxidative stress markers, the acute administration of BH_4_ has been shown to improve endothelial function in patients with SSc, without affecting their blood pressure (170). However, in this paper the authors did not investigate whether endothelial, rather than systemic, oxidative stress was ameliorated. Indeed, it may be possible that the concentration of BH_4_ used was sufficient to improve endothelial function but not appropriate to ameliorate the oxidative insult seen in blood samples of SSc patients. More importantly, the patients only received BH_4_ for 5 h. This period might be too short to influence systemic oxidative parameters. In fact, more data support the involvement of BH_4_ in SSc-associated endothelial dysfunction. For instance, circulating concentrations of BH_4_ have been found to be lower in plasma and pulmonary arteries of patients with IPF, and in rats with bleomycin-induced pulmonary fibrosis, when compared to their healthy counterparts (172). Notably, TGβ1 and ET-1 were able to induce EndMT in human pulmonary artery endothelial cells by decreasing BH_4_ and eNOS expression. Finally, treatment with sepiapterin, a BH_4_ precursor, blunted bleomycin-induced pulmonary fibrosis, ameliorated vascular remodeling *in vivo* by increasing plasma BH_4_ and vascular eNOS expression in rats and counteracted TGβ1- and ET-1-induced EndMT in human pulmonary artery endothelial cells *in vitro* (172).

In models of SSc-like bleomycin (BLM)-induced fibrosis, BLM-induced expression of collagen (I and III) synthesis mediated by ROS (173). Antioxidants such as NAC were found to attenuate BLM-induced lung fibrosis in mice and rats (174, 175) as well as collagen expression (173). The above data are consistent with earlier findings showing higher ROS production in type II alveolar epithelial cells and lung phagocytes in a rat model of BLM-induced fibrosis (176). Although the above-mentioned studies did not explore the involvement of EndoMT in the observed SSc-associated fibrotic process induced by ROS, recent evidence supports the role of ROS in promoting EndoMT in association with other pathological fibrotic conditions in the kidney (22, 177, 178). Furthermore, antioxidants have been shown to reduce EndMT of vascular endothelial cells (177). Indeed, suppressing oxidative stress has been shown to reduce *in vivo* EndMT in glomerular endothelial cells (22).

The BLM-induced fibrosis model has also been used in other studies (107, 179–181). Notably, Qi et al. (181) demonstrated that EndMT in the BLM-induced scleroderma mouse model is inhibited by geniposide, a constituent of the Chinese herbal compound Zhizi. According to Chinese herbal medicine, its “bitterness and coldness” properties are appropriate to attenuate various inflammatory conditions, including the early inflammation stage of SSc (181). The study proved the ability of Geniposide to block BLM-mediated EndMT not only *in vivo* (mouse model) but also *in vitro* (HUVECs). Further investigation of the EndMT-inhibiting effects exerted by Geniposide showed its ability to down-regulate key transcription factors involved in EndMT (Slug, Snail, Twist). Although the above-mentioned study does not report the involvement of oxidative stress, it is important to emphasize the well-known prooxidant effect of BLM which can damage surrounding cells, resulting in fibroblast activation (182). Interestingly, among all the pharmacological properties of Geniposide, its antioxidant activity was highlighted as protective in preventing cells from undergoing oxidative damage via MAP kinase pathway (183). Of note, ROS and EndMT interact through TGF-β-dependent and -independent pathways, as previously discussed. Based on the reported data, one could hypothesize that Geniposide might act as an anti-oxidant and anti-inflammatory factor, promoting an inhibitory effect on BLM-induced EndMT in the SSc mouse model. Although this hypothesis requires further investigations, a recent study demonstrated that salvianolic acid A (SSA), a natural polyphenol antioxidant, prevented EndMT both *in vitro* and *in vivo* by inhibiting oxidative stress (184). In *vitro*, EndMT was induced in human pulmonary arterial endothelial cells by TFG- β treatment, while *in vivo* EndMT was studied in a rat model of monocrotaline-induced pulmonary hypertension. SSA, by reducing ROS concentrations, was able to counteract EndMT-associated cell functions, signaling, and proteins both *in vitro* and in the lung of rats with pulmonary hypertension (184).

## Concluding remarks and future direction

SSc represents a public health and economic burden with a high rate of mortality and morbidity. ROS play a critical role in the pathogenesis of SSc. Both oxidative stress and EndMT are involved in the onset and progression of the fibroproliferative vasculopathy and fibrotic process in SSc. TGF-β is involved in the fibrotic process through EndMT, where ROS mediate and/or activate TGF-β to induce EndMT. Although numerous studies demonstrated the potential involvement of both ROS and EndMT in the development of fibrosis, only a few studies investigated the potential relationship between ROS and EndMT, and their involvement in the pathogenesis of SSc. The potential role of oxidative stress in inducing EndMT directly, or through the TGF-β-dependent pathway, is an emerging area of investigation that needs to be addressed. Elucidating the mechanism of SSc pathogenesis associated with ROS will contribute to the identification of therapeutic strategies to alleviate the costs and health burden of this disease.

## Author contributions

DT and GP ideated the review. DT wrote the first draft. HZ, AE, HA-S, GN, AM, and GP contributed to the editing and review of the different versions. GP and AM performed the final editing and GP submitted the manuscript.

### Conflict of interest statement

The authors declare that the research was conducted in the absence of any commercial or financial relationships that could be construed as a potential conflict of interest.

## Abbreviations

4-HNE: 4-hydroxynonenal, a common byproduct of lipid peroxidation during oxidative stress
ADAM17/NOTCH: Disintergrin and metalloproteinase domain-containing protein 17 involved in the activation of the Notch signaling pathway
AECA: Anti-endothelial cell antibodies
ALK5: Activin receptor-like kinase 5
BH4: Tetrahydrobiopterin
BLM: Bleomycin
c-Abl: c-Abl protein kinase
CTGF: Connective tissue growth factor
EMT: Epithelial-to-Mesenchymal Transition
EndMT: Endothelial-to-Mesenchymal Transition
ERK: Extracellular signal-regulated kinases
ET-1: Endothelial-1
FSP-1: Fibroblast specific protein-1
GSK-3β: Glycogen synthase kinase 3β
GTPase: Guanosine triphosphate intracellular signaling
HOCl: Hypochlorous acid
HPASMCs: Human pulmonary artery smooth muscle cells
I-EndMT: Induced EndMT
IL-1β: Interleukin-1β
miRNAs: MicroRNAs
MMP: Matrix metalloproteinase-1
NAC: N-Acetyl-Cysteine
NADPH oxidase (NOX): Nicotinamide adenine dinucleotide phosphate oxidase
NF-κB: Nuclear factor kappa-light-chain-enhancer of activated B cells
NO: Nitric oxide
${\text{O}}_{2}^{\cdot -}$: Superoxide radical
OH: Hydroxyl radical
ONOO^−^: Peroxynitrite
p38MAPK: P38 Mitogen-activated protein kinases
PAH: Pulmonary artery hypertension
PAI-1: Plasmin activator inhibitor 1
PDGF: Platelet derived growth factors
PDGFR: Platelet derived growth factor receptor
PECAM 1: Platelet endothelial cell adhesion molecule-1
PI3K: Phosphoinositide 3-kinase
PKC-δ: Protein kinase Cδ
PTU: Propylthiouracil
Ras: A small GTP-binding protein
ROS: Reactive oxygen species
SSc: Systemic sclerosis
TGF-β: Transforming growth factor-β
TIMPs: Tissue inhibitors of metalloproteinases
TNF-α: Tumor necrosis factor-alpha
VE-Cadherin: Vascular endothelial cadherin
VEGF: Vascular endothelial growth factor
vWF: Von Willebrand factor
α-SMA: α-smooth muscle actin.
